# Feasibility of Onchocerciasis Elimination with Ivermectin Treatment in Endemic Foci in Africa: First Evidence from Studies in Mali and Senegal

**DOI:** 10.1371/journal.pntd.0000497

**Published:** 2009-07-21

**Authors:** Lamine Diawara, Mamadou O. Traoré, Alioune Badji, Yiriba Bissan, Konimba Doumbia, Soula F. Goita, Lassana Konaté, Kalifa Mounkoro, Moussa D. Sarr, Amadou F. Seck, Laurent Toé, Seyni Tourée, Jan H. F. Remme

**Affiliations:** 1 Ministère de la Santé et de la Prévention Médicale, Dakar, Senegal; 2 Direction Nationale de la Santé, Bamako, Mali; 3 Multi-disease Surveillance Centre, Ouagadougou, Burkina Faso; 4 Université Cheikh Anta Diop, Dakar, Senegal; 5 Consultant, Ornex, France; Imperial College Faculty of Medicine, United Kingdom

## Abstract

**Background:**

Mass treatment with ivermectin is a proven strategy for controlling onchocerciasis as a public health problem, but it is not known if it can also interrupt transmission and eliminate the parasite in endemic foci in Africa where vectors are highly efficient. A longitudinal study was undertaken in three hyperendemic foci in Mali and Senegal with 15 to 17 years of annual or six-monthly ivermectin treatment in order to assess residual levels of infection and transmission and test whether ivermectin treatment could be safely stopped in the study areas.

**Methodology/Principal Findings:**

Skin snip surveys were undertaken in 126 villages, and 17,801 people were examined. The prevalence of microfilaridermia was <1% in all three foci. A total of 157,500 blackflies were collected and analyzed for the presence of *Onchocerca volvulus* larvae using a specific DNA probe, and vector infectivity rates were all below 0.5 infective flies per 1,000 flies. Except for a subsection of one focus, all infection and transmission indicators were below postulated thresholds for elimination. Treatment was therefore stopped in test areas of 5 to 8 villages in each focus. Evaluations 16 to 22 months after the last treatment in the test areas involved examination of 2,283 people using the skin snip method and a DEC patch test, and analysis of 123,000 black flies. No infected persons and no infected blackflies were detected in the test areas, and vector infectivity rates in other catching points were <0.2 infective flies per 1,000.

**Conclusion/Significance:**

This study has provided the first empirical evidence that elimination of onchocerciasis with ivermectin treatment is feasible in some endemic foci in Africa. Although further studies are needed to determine to what extent these findings can be extrapolated to other endemic areas in Africa, the principle of elimination has been established. The African Programme for Onchocerciasis Control has adopted an additional objective to assess progress towards elimination endpoints in all onchocerciasis control projects and to guide countries on cessation of treatment where feasible.

## Author Summary

The control of onchocerciasis, or river blindness, is based on annual or six-monthly ivermectin treatment of populations at risk. This has been effective in controlling the disease as a public health problem, but it is not known whether it can also eliminate infection and transmission to the extent that treatment can be safely stopped. Many doubt that this is feasible in Africa. A study was undertaken in three hyperendemic onchocerciasis foci in Mali and Senegal where treatment has been given for 15 to 17 years. The results showed that only few infections remained in the human population and that transmission levels were everywhere below postulated thresholds for elimination. Treatment was subsequently stopped in test areas in each focus, and follow-up evaluations did not detect any recrudescence of infection or transmission. Hence, the study has provided the first evidence that onchocerciasis elimination is feasible with ivermectin treatment in some endemic foci in Africa. Although further studies are needed to determine to what extent these findings can be extrapolated to other areas in Africa, the principle of onchocerciasis elimination with ivermectin treatment has been established.

## Introduction

Onchocerciasis control strategies have evolved significantly over the last three decades. The Onchocerciasis Control Programme in West Africa (OCP) [1], launched in 1975, used aerial larviciding of vector breeding sites in river rapids. This strategy was very successful in interrupting onchocerciasis transmission and ultimately eliminating the disease as a public health problem in the savanna areas of 10 West African countries [2]. However, aerial larviciding was not considered feasible or cost-effective elsewhere in Africa and in the absence of a drug that could be safely used in mass treatment, nothing was done to fight this debilitating disease in the rest of the continent where over 85% of the 37 million infected persons lived [3].

This situation changed dramatically in 1987 with the registration of ivermectin for the treatment of human onchocerciasis, and its donation free of charge for as long as needed by the manufacturer of the drug [4]. This revolutionized the fight against the disease, and led to the creation of the African Programme for Onchocerciasis Control (APOC) [5] that covered all the remaining onchocerciasis endemic areas in Africa, and the Onchocerciasis Elimination Programme for the Americas (OEPA) [6]. Currently, onchocerciasis control is nearly exclusively based on annual or six-monthly ivermectin treatment of all eligible members of communities at risk.

By the time APOC was launched in 1995, it was known from clinical and community trials that ivermectin was highly effective against the microfilariae that cause the severe manifestations of the disease, and hence that mass treatment with ivermectin was an effective strategy for controlling the disease as a public health problem [7]–[9]. But research had also shown that the drug had limited effect on the viability and productivity of the adult onchocercal worms which resumed production of microfilariae a few months after treatment [10], making it necessary to repeat treatment at intervals of no longer than one year to maintain microfilarial loads below levels of public health concern. Community trials had shown that mass treatment with ivermectin significantly reduced but did not interrupt onchocerciasis transmission during the first years of treatment, and given the adult worm life expectancy of about 10 years on average, it was concluded that annual treatment needed to be continued for a very long period of time [11]. Hence APOC's principal aim was to establish and sustain high treatment coverage in all areas where onchocerciasis was a public health problem [12]. To achieve this, APOC supported the establishment of community-directed treatment with ivermectin (CDTi) in all APOC countries [13],[14].

However, the question of whether, and if so when, the parasite could ultimately be eliminated with ivermectin treatment, and treatment safely stopped, remained unanswered at that time. Initial computer simulations with the model ONCHOSIM that were based on the results of the first community trials of ivermectin and the assumption that ivermectin is only a microfilaricide, predicted that annual treatment may needed to be continued for more than 25 years [11]. When subsequent studies after five years of treatment indicated that ivermectin treatment also reduced the fertility of the adult worms by some 30% after each treatment, these predictions were revised downward [15],[16]. However, this cumulative reduction in adult worm reproductivity was not seen in another study [17] and the predictions remained untested. Although it was generally believed that elimination would be possible in most of the Americas where onchocerciasis foci are often small and circumscribed, and several (though not all) vector species are relatively inefficient, there remained considerable uncertainty as to whether ivermectin treatment could ever achieve sustained interruption of transmission in Africa where onchocerciasis is endemic over vast areas and where all vectors are highly efficient [18]–[20].

Among the areas where large-scale ivermectin treatment was first introduced in Africa were onchocerciasis foci in Mali and Senegal in the Western Extension area of the OCP where treatment started in 1988 and 1989, shortly after the registration of ivermectin for the treatment of human onchocerciasis in 1987. Although part of the OCP, vector control was never used in this section of the Western Extension area and ivermectin has been the sole intervention tool since the start of control. A detailed review in 2001 of the available evidence on the impact of ivermectin treatment on onchocerciasis transmission in West and Central Africa showed that the prevalence of infection had fallen to very low levels after 12 years of treatment in onchocerciasis foci in Mali and Senegal in the Western extension area of the OCP [21]. The long period of treatment and the observed decline in prevalence of infection suggested that these foci would be among the first areas where the hypothesis of whether onchocerciasis can be eliminated with ivermectin from endemic foci in Africa could be tested. A longitudinal study was therefore started in 2005 in three initially hyperendemic onchocerciasis foci in Mali and Senegal to undertake a detailed assessment of the residual levels of infection and transmission, and, if sufficiently low, test whether ivermectin treatment could be safely stopped. The first results of this study are reported here.

## Methods

### Study sites

**Figure 1:**
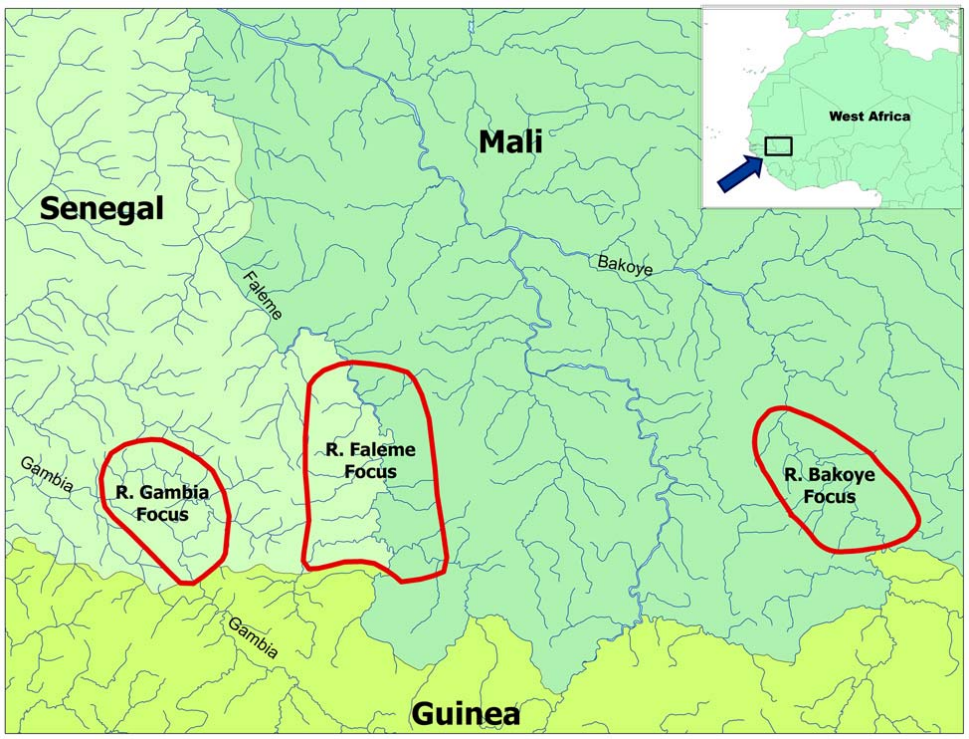
Location of the three study areas in Mali and Senegal.

The three study areas are located along the River Bakoye in Mali, the River Gambia in Senegal, and the River Faleme on the border of the two countries (figure 1). The study areas were selected on the basis of the following criteria: (i) they were part of the Western Extension area of the OCP where onchocerciasis control has been exclusively based on ivermectin treatment; (ii) ivermectin treatment started in 1988–1989 and the area was part of the first large-scale ivermectin treatment programs launched after registration of the drug in 1987; (iii) there existed good epidemiological baseline data for at least 10 villages where pre-control skin snip surveys had been undertaken by the OCP using standard onchocerciasis survey methods; (iv) the area contained hyperendemic villages, i.e. villages with a prevalence of microfilaridermia ≥60% or a Community Microfilarial Load (CMFL, the geometric mean number of microfilariae per skin snip among adults aged 20 years and above) >10 microfilariae per skin snip (mf/s) [22]–[24]; (v) the area was located along a river with known breeding sites of *Simulium damnosum* s.l., and has a length of at least 100 kilometers along the river and a width of at least 15 km at each side of the river. All three selected study areas met these criteria. An additional reason for including the River Gambia area was that it was the only area in Africa where six-monthly treatment with ivermectin had been given for more than 10 years. Demographically, the three study areas were similar with a rural population in 2006 of 20,000 to 30,000 people living in 75 to 94 villages per site (table 1). In the R. Gambia focus there is also one town with a population of about 18,000 but there are no urban settlements in the other two study areas.

**Table 1:** Ivermectin treatment history in the three study areas.

	River Gambia (6-monthly Rx*)	River Bakoye (annual Rx)	River Faleme (annual Rx)
**Geographic treatment coverage (year/no. villages treated)**			
First year with ivermectin treatment	1988/20	1989/28	1989/6
First year with all 1^st^ line villages treated	1990/30	1992/66	1991/61
First year with all villages treated	1992/79	1993/72	1993/87
Last year with all villages treated**	2006/83	2006/75	2006/94
Total years of treatment of all 1^st^ line villages	17 years	15 years	16 years
**Therapeutic coverage in treated villages**			
1988 to 1991	64%–69%	59%–62%	63%–68%
1992 to 1996	76%–77%	75%–78%	77%–81%
1998 to 2006	77%–81%	73%–83%	79%–89%

*Annual treatment in 1988 and 1989; 6-monthly from 1990 onwards.

**Increase in number of villages due to establishment of new villages.

**Figure 2:**
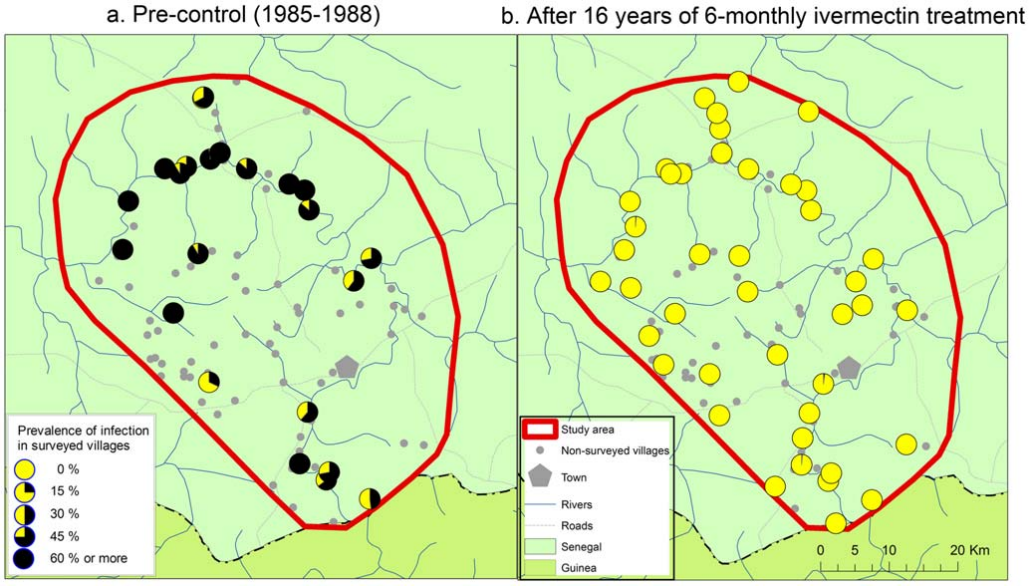
Prevalence of onchocerciasis infection in the R. Gambia focus.

**Figure 3 pntd-0000497-g003:**
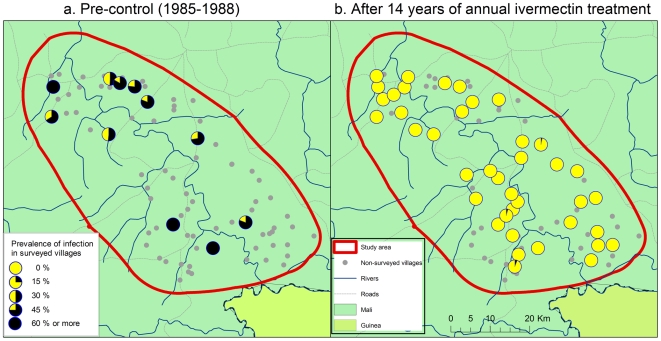
Prevalence of onchocerciasis infection in the R. Bakoye focus.

**Figure 4:**
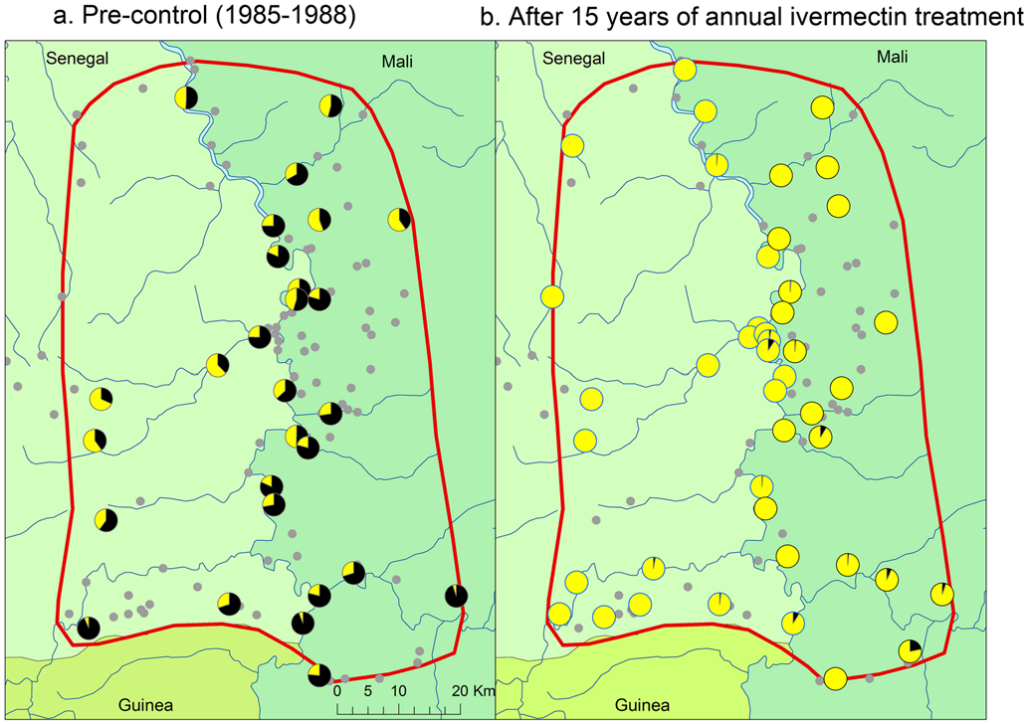
Prevalence of onchocerciasis infection in the R. Faleme focus.

De Sole et al. [25],[26] have mapped the pre-control distribution and severity of onchocerciasis in the Western Extension of the OCP, including all of Senegal and western Mali. According to their results, the selected study areas along the River Gambia and the River Bakoye were the two areas with the highest level of onchocerciasis endemicity in Senegal and western Mali where there was a high risk of onchocercal blindness. Along the River Faleme there was also an appreciable risk of onchocercal blindness along the southern part of the river where the study site is located. All three study sites were mapped in detail by the OCP and figures 2a, 3a and 4a show for each of the sites the spatial distribution of the prevalence of infection before the start of control. Onchocerciasis was endemic throughout the study areas, and in each area there were several hyperendemic villages. In the River Gambia focus, 8 out of 22 surveyed villages had a CMFL>10 mf/s (range 12.0 to 48.1 mf/s) [26]. In the River Bakoye focus 5 out of 11 surveyed villages had a CMFL>10 mf/s (range 10.2 to 21.6 mf/s) and in the River Faleme focus this was the case for 3 out of 27 surveyed villages, which had CMFL's of 13.3, 16.0 and 21.0 mf/s respectively.

All three onchocerciasis foci are isolated with respect to long-distance migration of the *Simulium* vectors except for the first few weeks of the rainy season. During the dry season, the rivers do not flow and there are no blackflies. At the beginning of the rainy season, when the Inter-tropical-conversion-zone (ITCZ) moves to the north, the breeding sites are reinvaded by simuliids from the south (mainly *S. sirbanum*) that migrate with the prevailing winds and start the repopulation of the breeding sites [27]–[29]. After a few weeks, when the winds change, this long distance migration stops and the vector population becomes purely local with virtually no migration from outside or from neighboring river basins. At the end of the rainy season, reverse migration takes place with blackflies from the study sites moving with the winds to perennial rivers in the south. All river basins involved in this migration pattern are either free from onchocerciasis or under large-scale ivermectin treatment since 1990. For the R. Bakoye, *S. dieguerense* has also been reported but this is a non-migratory *Simulium* species that only plays a local role in onchocerciasis transmission [30].

The three study areas are not completely isolated from neighboring endemic areas. Along all three rivers there are onchocerciasis endemic villages downstream of the study areas but their endemicity levels are generally lower and they are all covered by the same national ivermectin treatment programs of Mali and Senegal. The neighboring river basins are also endemic for onchocerciasis and undergoing ivermectin treatment. Although there is little vector migration between the river basins, human migration cannot be excluded. Upstream in Guinea there are some endemic areas that are also reported to be under ivermectin treatment. Hence, the three study areas cannot be considered completely isolated areas, but rather as the most endemic sections of onchocerciasis zones along three rivers that are fully covered by the national ivermectin treatment programs.

### Ivermectin treatment history

Ivermectin treatment started first in 1988 in the R. Gambia focus as part of the community trials of ivermectin undertaken by the OCP to confirm the safety of large-scale ivermectin treatment [31], and in 1989 in the other two foci (table 1). Treatment was not immediately introduced in all villages in the three areas but first targeted at the most affected villages. During the next 5 years the treatment program was gradually expanded until it covered all villages. As a result of this stepwise introduction of treatment, the number of years that each village had received treatment by the time of the study ranged from 14 to 19 years. From an epidemiological point of view, the most significant period was when all first-line villages, located near the river and the vector breeding sites and which play a dominant role in onchocerciasis transmission [32], were treated. This was achieved for the R. Gambia from 1990 onwards, for the R. Bakoye from 1992 onwards and the R. Faleme from 1991 onwards. Hence, by the end of 2006, all first-line villages in the R. Gambia area had been under treatment for 17 years, in the R. Bakoye area for 15 years, and in the R. Faleme area for 16 years. We will use those numbers when referring to the number of years of ivermectin treatment in each study area. In the R. Gambia focus, treatment was given at six-monthly intervals from 1990 onwards, and the number of treatments per village ranged from 30 to 36, with all first-line villages receiving at least 34 treatments. The urban area was excluded from treatment in accordance with national treatment policy. In the other two basins treatment was given annually in all villages.

Initially, ivermectin treatment was ensured by mobile teams of the Ministry of Health. The reported treatment coverage during the first three years was not very high but subsequently improved and reached between 75 to 81% of the total population between 1992 and 1996. In 1997, there was a change in policy and treatment was changed from the costly mobile-team approach to Community-directed Treatment with ivermectin (CDTi) [2]. The new policy was introduced rather abruptly while there was some resistance from health workers who would no longer benefit from the financial support that OCP provided for mobile teams. As a result, there was a fall in treatment coverage during the transition year of 1997. In 1998, the situation was corrected and following proper social mobilization efforts, CDTi took off effectively. A second implication of the change from mobile teams to CDTi was the integration of treatment reporting into the national health information systems. This was initially problematic and for several years the available records were incomplete (and largely missing for 1997) until the new system was properly functioning. The change to CDTi resulted in a further improvement of treatment coverage which in several years even exceeded 80% of the total population (about 95% of eligibles). Overall, the reported treatment coverage has been high since 1992 with the exception of the year 1997.

### Study design

Onchocerciasis elimination is here defined as the reduction of local onchocerciasis infection and transmission to such low levels that transmission can no longer sustain itself and treatment can be safely stopped without risk of recrudescence of infection and transmission. Surveillance would still be needed to detect possible reintroduction of the parasite through human or vector migration from other endemic areas where elimination has not yet been achieved.

**Figure 5:**
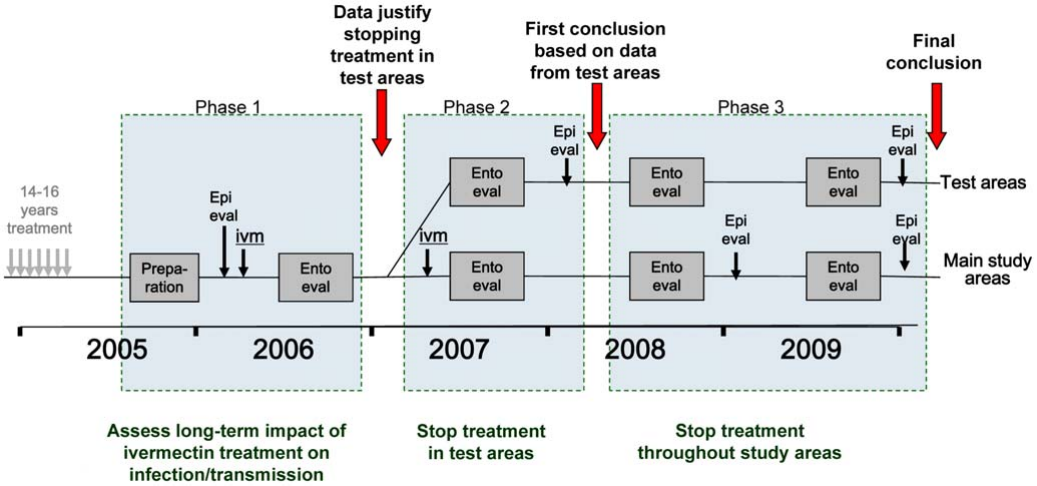
Study design and study phases in each study site.

To assess whether elimination has been achieved in the three study areas, the study was designed in three phases (figure 5). The aim of the first phase was to undertake a detailed assessment of onchocerciasis infection and transmission levels after 14 to 17 years of treatment. Skin snip surveys were to be undertaken in a stratified random sample of some 40 villages in each study site, and transmission would be monitored for a full transmission season through entomological evaluations in 4 to 6 fly-catching points per study site. If the observed infection and transmission levels in a study site were below predefined, provisional thresholds (see section on indicators below), phase 2 would start in which treatment would be stopped in a test area of 5–8 villages located around one of the catching points in the study site. The effect of stopping treatment on infection and transmission would be evaluated by epidemiological surveys 20 to 22 months after the last treatment in the test villages, and by entomological evaluation in all catching points during another full transmission season. If there was no recrudescence of infection and transmission in the test area, phase 3 would start in which treatment would be stopped throughout the study site and infection and transmission monitored for another two years in all sample villages and catching points. The first two phases of the study have been completed in all three study sites.

### Epidemiological evaluation methods

At the beginning of the study, all villages located in the study area were visited to obtain exact geographic coordinates using a geographic positioning system (GPS). These coordinates were used to generate exact maps of the study areas, and using these maps a spatial sample of at least 40 villages were selected to be surveyed during the first phase of the study. Of these 40 villages, 20 were selected from the first-line villages along the river, while ensuring a good spatial coverage along the length of the river basin, and the remaining 20 villages selected randomly from the second line or further away from the breeding sites. Skin snip surveys were done in all selected villages 11–12 months after the last treatment round. A few selected villages proved to be very small (<50 people), and for those the nearest village was also included in the surveys.

In each village, all persons above the age of 1 year who agreed to participate (or whose parent agreed for them to participate in the case of children) were examined for onchocerciasis infection. The surveys used established skin snip examination methods in which the national onchocerciasis teams have been trained in the past by the OCP. Two skin snips were taken from the iliac crests with a 2 mm Holth corneoscleral punch and microscopically examined after incubation for 30 minutes in distilled water (and a further 24 hours in saline for negative skin snips) for the presence and number of *O. volvulus* microfilariae [33]. The numbers of microfilariae were counted and the results recorded for each person examined. Basic information on the migration history for each person during the last 10 years before the survey was also collected.

During phase 2, treatment was stopped in test areas of 5–8 villages located around one of the catching points. Skin snip surveys were done in all test villages 20–22 months after the last treatment in 2006. During these surveys, an additional diagnostic test was also used. This was an improved version of the traditional diethylcarbamazine-citrate (DEC) patch test [34] that had recently been developed by LTS Lohmann Therapie-Systeme AG and undergone successful clinical testing at the Onchocerciasis Chemotherapy Research Centre in Hohoe (OCRC), Ghana (K. Awadzi, personal communication). The new test uses transdermal technology for the application of a low dose of 5.4 mg of DEC-citrate on the skin which produces within 24 hours a characteristic skin reaction in persons infected with *O. volvulus*. The new patch test was applied at the same time as the skin snip examination. Patients were requested to return 24 hours later when the patch was removed and the skin examined. A positive skin reading was defined as the presence of a characteristic skin lesion consisting of mild edema of the area covered by the patch, studded by fine pinpoint papules. Before the surveys, all examiners were trained by a senior technician from OCRC in the application of the DEC patch test, and in standardized reading of skin reactions.

### Entomological evaluation of onchocerciasis transmission

During each phase, a detailed entomological evaluation was done throughout the full transmission season in order to determine the levels of *O. volvulus* transmission. Four vector catching points were selected per study area (six for the river Faleme which covers a larger area and in two countries). Every week, 3 days of capture were carried out at each catching point during the transmission period which generally covers 4 to 5 months per year (June–October or July–October). Flies were collected using the method of bulk catches with a team of 3 to 4 fly catchers working from 7 AM to 6 PM. Each daily catch was preserved in 80% alcohol and sent to the DNA laboratory of the Multi-Disease Surveillance Centre (MDSC) in Ouagadougou, Burkina Faso [35]. In the laboratory, the flies were rinsed with distilled water, the heads separated from the bodies and sorted in lots for DNA extraction. The purified DNA was used as a substrate in a O-150 (an *Onchocerca*-specific DNA sequence) PCR, and the resulting product classified by hybridization to the *O. volvulus*-specific oligonucleotide probe OVS-2 [36],[37]. A computer program (Poolscreen™) was used to translate the molecular biology data obtained from screening pools into an estimate of the infectivity rate in the vector population [37].

### Indicators

The two main indicators of onchocerciasis infection and transmission used in the present study are the vector infectivity rate as measured by the number of flies with *O. volvulus* L3 (infective) larvae in the head per 1,000 flies (FLH/1,000) and the prevalence of microfilariae in the skin in the human population. Model predictions as well as large-scale experience in the OCP had shown that these indicators do not have to be equal to zero to ensure elimination, but that there are thresholds below which infection and transmission will die out [38]–[40]. Computer simulations with the model ONCHOSIM predicted that the risk of recrudescence was negligible if the vector infectivity rate was below 0.9 to 1.3 FLH per 1,000 parous flies and the OCP adopted therefore a threshold of 1 FLH per 1,000 parous flies [41]. When after 14 years of vector control, onchocerciasis elimination appeared to have been achieved in the original OCP area, it was decided to stop vector control operations in nine river basins. To ensure that the decision to stop had been correct, a large scale entomological evaluation was undertaken during the first two years after stopping vector control [39]. The results showed that there were still infective flies in each river basin but at levels below the threshold of 1 FLH per 1,000 parous flies. Definite evidence that the decision to stop vector control had been correct was provided by epidemiological surveys undertaken 10 years after the cessation of control which showed that there had been no recrudescence of infection [38],[42].

The entomological evaluation methods used by the OCP involved dissection of hundreds of thousands of flies, which was technically and financially highly demanding and difficult to sustain by the countries alone after the closure of the OCP in 2002. When pool screening became operational in 1998, it was made the standard method for entomological surveillance of onchocerciasis transmission by national onchocerciasis control programs in the OCP countries, supported by the MDSC [43]. In this approach, black flies are collected by village members for the full transmission season and subsequently forwarded through the national onchocerciasis control programs to the MDSC molecular biology laboratory in Ouagadougou for analysis [44]. As no fly dissections are done in the field, the proportions of parous flies are not known. The threshold of 1 FLH per 1000 parous flies was therefore converted by the OCP to a threshold of 0.5 FLH per 1,000 flies, assuming an average parous rate of about 50% over the transmission season [45]. The pool screen method and the corresponding threshold appear to have worked well for entomological surveillance in West Africa since 1998, confirming that transmission levels remained insignificant in most river basins but having detected residual transmission in a few areas where control was known to have been unsatisfactory. The same standard pool screening method with a pool size of 300 flies and threshold of 0.5 FLH per 1,000 flies were used in the current study. To ensure that a sample with 0 FLH would imply that the infectivity rate was with 95% confidence below the threshold of 0.5 FLH per 1000 flies, a minimum of 3900 flies was to be analyzed per catching point [37].

The provisional thresholds for the prevalence of infection in the current study were also defined on the basis of the experience with successful cessation of vector control in the OCP. Just before stopping control, the OCP had undertaken skin snip surveys in eleven initially hyperendemic villages from the nine river basins. Four of the villages had become skin snip negative but seven villages still had a prevalence of infection between 1.0% and 4.8% [39]. Guided by these data, the provisional thresholds for elimination with ivermectin treatment in the present study were conservatively defined as a microfilarial prevalence <1% in 90% of sample villages, and a prevalence <5% in 100% of sample villages.

The above thresholds were provisional thresholds to guide decision making and analysis in the current study. One of the objectives of the study is to review these thresholds, and revise them as required, in a detailed model-based analysis of the final study results.

### Research ethics

Ethical review and clearance of the research protocol, research instruments and informed consent procedures were obtained from the national ethical review boards of the ministries of health in Mali and Senegal, as well as from the World Health Organization (WHO) ethical review committee. Community meetings were held in all villages to explain the research objectives and procedures, and the right of each individual to decide whether to participate in the examinations or not. Before each examination, each individual who had voluntarily come to the examination point and agreed to participate signed, or put a thumb print if not literate, on the examination form to indicate consent. For children, one of the parents or the responsible guardian would sign the examination form. The use of community meetings to discuss the research project and the right of individuals to refuse participation in the examination was considered the most culturally appropriate and effective method for providing the necessary information to community members, and this approach was approved by both the national ethical review boards and the WHO ethical review committee.

## Results

### Onchocerciasis infection and transmission after 14 to 17 years of ivermectin treatment

During phase 1, epidemiological evaluations were done in 126 villages between mid March to mid May 2006, just before the last full treatment round in April and May 2006 (table 2). A total of 17,890 persons (71.1% of the census population) voluntarily came to the examination points and agreed to participate in the skin snip examination. Those who did not participate included 11.3% of the census population who were absent from the village for up to one year, and 17.6% who were in the village but did not come to the examination for reasons of non-eligibility (age<1 year), advanced age or illness, or who refused to participate. Information on refusal was obtained indirectly from family or other community members, indicating that some 9% of the census population refused to participate in the skin snip examination.

**Table 2:** Results of epidemiological evaluations after 14–16 years of treatment.

Study area	Ivermectin treatment	# villages surveyed	Census population	Skin snip examination				Percentage of survey villages with	
				Examined	Mf+ve	Prevalence	Geometric mean mf/s in mf+ves	Prev.mf<1%	Prev.mf<5%
R. Gambia	6-monthly	42	7,184	5,271	3	0.06%	2.3	98%	100%
R. Bakoye	Annual	40	9,868	6,899	18	0.26%	10.3	95%	100%
R. Faleme	Annual	44	8,106	5,720	48	0.84%	5.8	80%	91%

The results of the evaluations showed that 14 to 16 years of ivermectin treatment had fundamentally changed the epidemiological situation in all three study areas (figures 2 to [Fig pntd-0000497-g003] 4). While onchocerciasis was highly endemic during the pre-control period in the R. Gambia area, after 16 years of treatment only 3 out of 5,271 persons examined were skin snip positive and 98% of villages had a microfilarial prevalence <1%. A similar change was seen in the R. Bakoye where the prevalence in this previously hyperendemic focus had dropped to 0.26% and 95% of villages had a microfilarial prevalence <1%. It is noteworthy that 13 of the 18 skin snip positives in the R. Bakoye focus came from one third-line village. Further investigation revealed that the families concerned lived most of the year on their farms on the river banks, far away from their village but close to the vector breeding sites. Because of the distance to the village, most of them had never or only once been treated with ivermectin. Their skin microfilarial loads were generally low except for two persons, one male of 32 years who was never treated and one boy of 10 years who was treated once, and who had microfilarial loads of 87 mf/s and 96 mf/s respectively. Along the R. Faleme the epidemiological results were equally good in the north and in the center of the study area, with only 11 infected persons in 31 villages examined. However, in the southern third of the focus there were still seven villages with a microfilarial prevalence between 1% and 13%: 4 villages in Mali with a total of 15 infected persons and 3 villages in Senegal with 22 infected persons.

**Figure 6:**
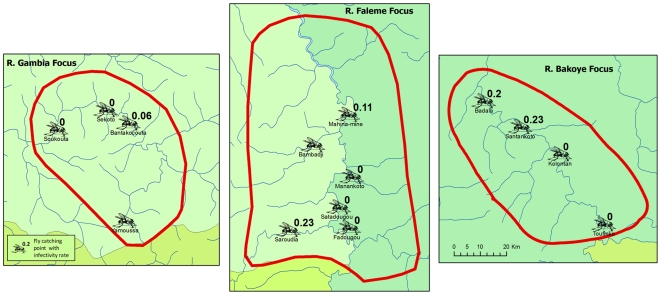
Vector infectivity rates in the three study areas after 15 to 17 years of ivermectin treatment.

Following the last full treatment round of early 2006 (and thus after 15 to 17 years of treatment), entomological evaluations of onchocerciasis transmission were undertaken during the rainy season from July to November 2006. The results are summarized in table 3. A total of 157,500 black flies were collected through the bulk catches method and examined in the molecular biology laboratory in Ouagadougou using the pool screening technique [37],[44]. For all catching sites the number examined exceeded the target of 3,900. The results showed that onchocerciasis transmission levels were extremely low in all three river basins. In seven of the catching points, not a single infective larva was detected. In the remaining five catching points, the vector infectivity rate was below the threshold of 0.5‰. The location of the catching points is shown in figure 6. Two catching points in Senegal, Yamoussa along the R. Gambia, and Bambadji along the R. Faleme, were for logistic reasons not yet operational in phase 1. For the others, figure 6 also shows the vector infectivity rate.

**Table 3:** Results of entomological evaluations after 15–17 years of treatment.

Study area	Country	Fly Catching Point	Number of flies examined	Infectivity rate (‰)	95% Confidence interval (‰)	
R. Gambia	Senegal	Bantacokouta	15,900	0.063	0.002	0.327
	Senegal	Sekoto	21,300	0	0	0.090
	Senegal	Soukouta	3,900	0	0	0.492
R. Bakoye	Mali	Badala	10,200	0.202	0.002	0.709
	Mali	Kolontan	5,400	0	0	0.355
	Mali	Tieourou	18,000	0.230	0.044	0.667
	Mali	Toufinko	12,000	0	0	0.150
R. Faleme	Mali	Fadougou	19,500	0	0	0.098
	Mali	Mahina Mine	18,300	0.110	0.013	0.390
	Mali	Manankoto	14,100	0	0	0.130
	Mali	Satadougou	14,400	0	0	0.130
	Senegal	Saroudia	4,500	0.229	0.007	1.180
**TOTAL**			157,500			

Both in the R. Gambia and the R. Bakoye areas all epidemiological and entomological indicators were below the provisional thresholds for elimination. In R. Faleme area, the epidemiological results for the center and north of the area were below the threshold, as were the infectivity rates for all catching points. Based on these results, it was decided to proceed with phase 2 of the study and stop treatment in test areas in each of the three study foci.

### Onchocerciasis infection and transmission after stopping ivermectin treatment in test areas

**Figure 7:**
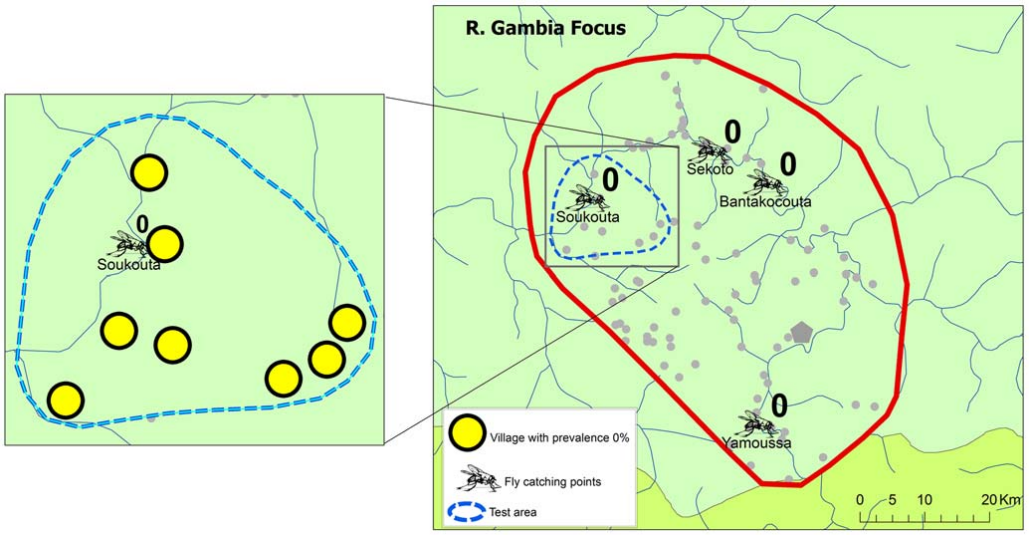
Vector infectivity rates and prevalence of infection 16 to 22 months after the last treatment in the test area in the R. Gambia focus.

Following the decision to proceed with the cessation of treatment, test areas were identified in each of the study areas (figures 7 to [Fig pntd-0000497-g008] 9). Each test area consisted of 5 to 8 villages located around a catching point, and included at least one village that had a skin snip positive person in the phase 1 surveys. Treatment was stopped in all villages in the test areas and during the next treatment round in 2007, ivermectin treatment was only given in the study villages outside the test areas. The impact of stopping ivermectin treatment on infection and transmission was evaluated by epidemiological surveys that were undertaken in January and February 2008, i.e. 20 to 22 months after the last treatment in the test villages, and entomological evaluation in all catching points during the transmission season of 2007.

**Figure 8 pntd-0000497-g008:**
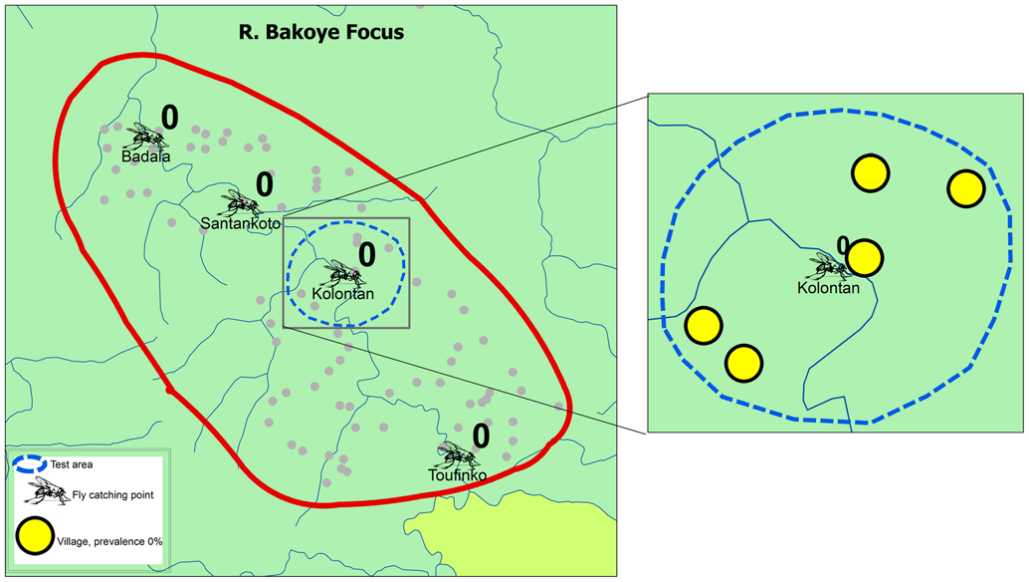
Vector infectivity rates and prevalence of infection 16 to 22 months after the last treatment in the test area in the R. Bakoye focus.

**Figure 9:**
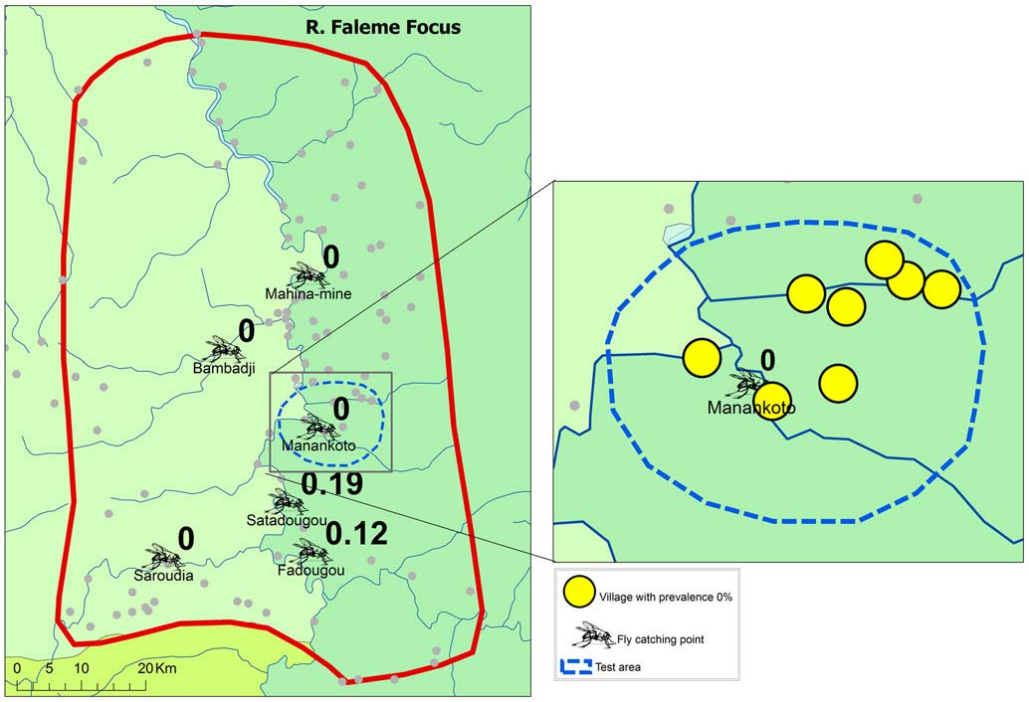
Vector infectivity rates and prevalence of infection 16 to 22 months after the last treatment in the test area in the R. Faleme focus.

The results of the epidemiological evaluation are summarized in table 4. This time only 55% of the census population came voluntarily to be examined, 22% were absent from the village, 5% were not eligible or could not come because of advanced age or illness, and 28% of the population refused to be examined.

**Table 4:** Results of epidemiological evaluations 20–22 months after the last treatment in the test areas.

Study area	Ivermectin treatment	# villages surveyed	Census population	Skin snip examination			DEC patch test		
				Examined	Mf+ve	Prevalence	Examined	+ve	Prevalence
**R. Gambia**	6-monthly	8	1,136	775	**0**	0%	775	**0**	0%
**R. Bakoye**	Annual	5	2,188	1,066	**0**	0%	933	**0**	0%
**R. Faleme**	Annual	8	827	442	**0**	0%	408	**0**	0%

A total of 2,283 people were examined in 21 test villages, and all of them were skin snip negative. The same result was obtained with the DEC patch test for which also everybody was negative in all three study sites. The numbers examined with the DEC patch test are lower than those with the skin snip method in two of the study areas because of people not returning after 24 hours for the follow-up examination. Furthermore, up to one third of the patches had partly or completely detached during the 24-hour follow-up period. The few persons who were skin snip positive during phase 1 in these tests villages had become skin snip negative or could not be examined because of their absence from the village.

The entomological evaluation was done from mid August 2007 to mid December 2007, i.e. 16 to 20 months after the last treatment in the test areas. Again, a very large number of 123,000 black flies was collected through bulk catches and examined in the molecular laboratory in Ouagadougou. For all but one catching points the number examined largely exceeded the target of 3,900 flies (table 5). The results showed that overall the vector infectivity rate was even lower than in phase 1.

**Table 5:** Results of entomological evaluations 16–20 months after the last treatment in the test areas.

Study area	Country	Fly Catching Point	Number of flies examined	Infectivity rate (‰)	95% Confidence interval (‰)	
R. Gambia	Senegal	Bantacokouta	8700	0	0	0.220
	Senegal	Sekoto	8400	0	0	0.228
	Senegal	Soukouta	10800	0	0	0.177
	Senegal	Yamoussou	6300	0	0	0.304
R. Bakoye	Mali	Badala	10800	0	0	0.177
	Mali	Kolontan	12300	0	0	0.156
	Mali	Tieourou	7220	0	0	0.266
	Mali	Toufinko	16800	0	0	0.114
R. Faleme	Mali	Fadougou	8400	0.121	0.003	0.624
	Mali	Mahina Mine	8400	0	0	0.228
	Mali	Manankoto	8400	0	0	0.228
	Mali	Satadougou	8100	0.186	0.0038	0.647
	Senegal	Saroudia	2700	0	0	0.711
	Senegal	Bambadji	5700	0	0	0.336
**TOTAL**			123000			

Figures 7 to [Fig pntd-0000497-g008] 9 show the location of the catching points and the surrounding villages in the test areas. The vector infectivity rates at the catching points in the test areas were zero in all three study sites, as well as in most other catching points. Only in two catching points in the R. Faleme focus were infective larvae detected but the infectivity rate was again below the threshold of 0.5 FLH/1,000.

In phase 2 all epidemiological and entomological indicators were below the provisional elimination thresholds, and it has therefore been decided to proceed with phase 3. Treatment has now been stopped in all villages in the R. Gambia and R. Bakoye study areas. Because of the less satisfactory epidemiological results in the southern part of the R. Faleme, it was decided to proceed more cautiously and create two new test areas in the southern section of the focus where treatment has been stopped first and will be evaluated for one year before a final decision is taken to stop treatment in all villages throughout the focus.

## Discussion

Ever since ivermectin became the principal tool for onchocerciasis control, it has been debated whether, in addition to controlling the disease as a public health problem, it could also be used to interrupt transmission and eliminate the parasite [11],[18]. As the drug does not kill or permanently sterilize the adult worms, elimination was clearly not possible in the short term. However, it was not unreasonable to assume that sustained interruption of transmission could be achieved after a long period of mass treatment. The first community trials had shown that mass treatment with ivermectin significantly reduces transmission and thus the incidence of infection with new worms [46]–[49]. It was likely, therefore, that repeated mass treatment would result in a progressive reduction in transmission of the parasite, probably accelerated by an additional effect of ivermectin treatment on the fertility of the adult worm [15]. Model predictions had indicated that elimination might be possible [16], but empirical longitudinal data were not yet available to test this prediction and there remained considerable uncertainty as to whether elimination could be achieved, especially in Africa where the disease is endemic over large areas and where the vectors are highly efficient [18]. The current study has provided the first evidence that elimination of onchocerciasis with ivermectin treatment is feasible in some endemic foci in Africa. After 15 to 17 years of annual or six-monthly ivermectin treatment in three foci in Mali and Senegal, only few infections remained in the human population, infective *O. volvulus* larva were extremely rare in hundreds of thousands black flies examined, and vector infectivity rates were everywhere below the postulated threshold for interruption of transmission.

The evidence generated after stopping treatment in test areas of each focus was even more convincing. Evaluations conducted 16 to 22 months after the last treatment showed no recrudescence of infection in the human population and no recrudescence of transmission. In fact, not a single skin snip positive person or infected black fly was detected in the test areas themselves. This is a significant finding as one of the main uncertainties was whether the residual adult parasite population was still sufficiently viable to restart microfilarial production after the withdrawal of ivermectin. The fact that no skin microfilariae were found up to 22 months after the last ivermectin treatment indicates that even if there still were adult worms in the human population, they were no longer productive or produced too few microfilariae, to be detected by the skin snip method, and posed therefore no significant risk for onchocerciasis transmission.

A difficulty in the present study was to define the total treatment period in each study area. The treatment programs were introduced in a stepwise manner, covering during the first years the most infected villages and gradually expanding coverage during subsequent years to villages with lower levels of endemicity. We have defined the effective treatment period as the number of years that all first-line villages were included in the treatment program. These villages are ‘first line’ towards the river with no other human populations between them and the vector breeding sites, and they play a dominant role in onchocerciasis transmission [32]. The implication of this definition is that some first-line villages received treatment for one or two more years than the overall treatment period reported, and other villages that were deemed less important for transmission received less years of treatment.

A unique feature of the current study is that it allowed a comparison of the long-term impact of two different treatment strategies: annual and six-monthly treatment. The final results in the R. Gambia focus, where ivermectin treatment was given at six-monthly intervals, and in the R. Bakoye, where treatment was annual, were virtually identical. The prevalence of infection had fallen to very low levels in both areas, the vector infectivity rates were close to zero and, most importantly, there had been no recrudescence in infection and transmission after stopping treatment in the test areas. In the R. Faleme focus, where treatment was annual, the evaluation results were equally good in the centre and north of the focus, but in the south there were still seven villages with a microfilarial prevalence between 1% and 13%. A higher prevalence in the south was seen both on the right bank of the river in Mali and on the left bank in Senegal, suggesting that the reason was of a spatial nature rather than related to treatment coverage or strategy. The R. Faleme borders on Guinea in the south and the results could be explained by some limited reinfection originating from across the border. The critical question for elimination, however, is whether the residual levels of infection in the R. Faleme constitute a risk for recrudescence of transmission. Vector infectivity rates were below the postulated threshold for elimination in all catching points in the R. Faleme focus, including in the south, and following cessation of treatment in the test area there was no evidence of recrudescence. It appears that elimination has also been achieved in the north and centre of the R. Faleme focus, and possibly in the south but this will be further investigated in 2009. From the perspective of elimination, therefore, the impact of 15–17 years of treatment was not very different between the three river basins and the six-monthly treatment regimen did not show a clear advantage over annual treatment. However, historical epidemiological evaluation data of the OCP have shown that infection levels in the R. Gambia initially fell much faster than in other river basins [21] and it is quite possible that elimination was achieved several years earlier in the area with six-monthly treatment.

Although the current study has provided the first evidence of elimination with ivermectin treatment in onchocerciasis endemic areas in Mali and Senegal, the results do not imply that elimination is feasible in all other endemic areas in Africa. The feasibility of elimination depends on several factors that may vary significantly between onchocerciasis endemic areas, e.g. pre-control endemicity levels, vector competence, human and vector migration, and treatment factors of coverage, frequency, duration, and efficacy.

Previous modeling studies have indicated that the probability of elimination of onchocerciasis infection and transmission depends strongly on the pre-control level of endemicity [16]. The endemicity level reflects the density and competence of the local vector population and the intensity of human-vector contact during the pre-control period, and it is therefore an important predictor of the local potential for transmission after cessation of treatment. In all three river basins there were initially hyperendemic villages and their maximum intensity of infection, as reflected by the CMFL, ranged from 22 to 48 mf/s. Although these fall within the hyperendemic range, there are many onchocerciasis foci in Africa where the level of endemicity is significantly higher, and where elimination will probably be more difficult to achieve.

*Simulium* species differ considerably in vector competence and elimination is predicted to be more difficult when vector competence is high [1],[50]. The importance of vector competence was already obvious during the first community trials of ivermectin which showed a much greater reduction in onchocerciasis transmission after ivermectin treatment in an onchocerciasis focus in Guatemala, where the vector *S. ochraceum* s.l. has a relatively low vector competence, than in community trials in Africa where the vectors belonged to the *S. damnosum* complex [46]–[48]. In the study areas in Mali and Senegal the main vector is *S. sirbanum*, which is the most widely distributed vector in West Africa and the predominant vector in the dry savanna. [1],[30],[51]. In the wet savanna the distribution of *S. sirbanum* overlaps with that of *S. damnosum* s.s. These two savanna species cannot be differentiated morphologically and there exist only few studies that have analyzed elements of vector competence for these two species separately, showing no consistent difference between *S. sirbanum* and *S. damnosum s.s.* [52],[53]

Transmission is seasonal in the study sites in Mali and Senegal and only takes place during the rainy season when the vectors have repopulated the breeding sites. Seasonal transmission does not necessarily imply less transmission than in areas where the vectors are present throughout the year. In fact, the reverse is often true in West Africa where the highest endemicity levels are found in areas with seasonal rather than perennial transmission. But seasonal transmission allows for a treatment strategy that optimizes the impact of annual treatment on transmission by distributing ivermectin just before the start of the rainy season. This ensures that microfilarial loads are at their lowest during the transmission season and that when they rise again there are no vectors around to ingest such microfilariae.

The above characteristics of the study sites, i.e. seasonal transmission by *S. sirbanum* and endemicity levels in the lower range of hyperendemicity with CMFL's between 10 and 20 mf/s, and occasionally up to 40–50 mf/s, are typical for the dry savanna belt in West Africa which runs from Senegal and Mali, through northern Nigeria to Chad and Sudan [1],[30],[51],[54]. This is a vast area with millions of people infected with onchocerciasis, for whom the study findings are directly relevant. However, more to the south the vectors are different and there are many areas where pre-control endemicity levels are higher and where elimination may be more difficult. There is therefore an urgent need for further investigations to determine to what extent the findings of the current study can be extrapolated to other onchocerciasis endemic areas in Africa.

Experience with vector control in the OCP has shown that long-distance migration by infected simuliids from outside a control program area can result in significant transmission within the area under control [27]. The study areas in Mali and Senegal were not subject to long-distance vector migration except for a short period at the beginning of the rainy season when a new wave of simuliids repopulated the breeding sites. However, the study foci were not completely isolated from neighboring endemic areas. Along the rivers there were other endemic villages beyond the boundaries of the study areas and all neighboring river basins were endemic for onchocerciasis. It is likely that there was some vector dispersal along the rivers across the study boundaries as well as some human movement from other endemic areas, but, with the possible exception of south Faleme, this did not result in any significant infection or transmission in the study areas. The reason is probably that all onchocerciasis endemic areas in Senegal and western Mali have been treated by the national ivermectin treatment programs of the two countries since the early 1990s, irrespective of whether they fell within or outside the boundaries of the current study, and that the epidemiological situation was equally good (if not better because of lower pre-control endemicity levels) outside the study areas. It is quite possible therefore that onchocerciasis is near elimination in all of Senegal and western Mali, and that nationwide elimination of onchocerciasis may be a realistic target for these countries for the coming years.

With the exception of the year 1997, annual treatment coverage was good throughout the control period and this is an important reason for the results obtained. Onchocerciasis foci where treatment coverage has been less good or where the geographic coverage has been patchy, may require considerably more years of treatment to achieve elimination. On the other hand, in this first experimental cessation of ivermectin treatment ever, we have proceeded very prudently and it is possible that equally satisfactory results might have been obtained if treatment had been stopped a few years earlier.

The study provided a unique opportunity to evaluate a new diagnostic test in the field. The improved DEC patch test was easy to use and was shown to be highly specific in these onchocerciasis foci in West Africa. A high specificity is critically important for the potential use of the test as an epidemiological tool in low prevalence situations. The DEC patch test had some operational shortcomings, i.e. the requirements to return for the examination 24 hours after application of the patch (due to the test measuring a delayed hypersensitivity reaction to microfilarial antigens) which led to the failure of some people to do so, and a considerable proportion of patches having become partly or completely detached during the 24-hour period. But these limitations do not outweigh the great advantage that the DEC patch test, as a noninvasive test, has over the skin snip examination in which the populations of endemic areas are increasingly reluctant to participate. This was also evident in the current study where a quarter of the population refused to participate in the skin snip examination during the second phase of the study. This high rate of refusals might have introduced some bias in the epidemiological evaluation, and it was therefore important to have a second, independent source of evidence on interruption of transmission from the entomological evaluation in the same locations.

The results of the study, indicating elimination after 15 to 17 years of annual or six-monthly ivermectin treatment, are quite consistent with previous ONCHOSIM predictions on the feasibility of elimination for comparable levels of endemicity, treatment coverage and treatment frequency [16]. These predictions were based on data from the first five years of ivermectin treatment only, and a more detailed model-based analysis of the data of the current study is being undertaken to develop improved predictions of where and when ivermectin treatment can be safely stopped. Furthermore, the thresholds for elimination used in the current study are provisional and based on previous model predictions and large-scale evaluations after cessation of vector control. A second objective for the ongoing modeling research therefore is to review and revise the elimination thresholds for ivermectin treatment on the basis of the data from the current study.

The study in Mali and Senegal still continues. Following the excellent results of the second phase, the third phase of the study has now been started and will generate additional data on onchocerciasis infection and transmission two to three years after stopping treatment in all villages in the three onchocerciasis foci. If the follow-up findings confirm the current results, they would provide the definite evidence that it was safe to stop ivermectin treatment and that onchocerciasis infection and transmission has been eliminated from the three foci in Mali and Senegal.

In the meantime, the study has provided the first evidence that onchocerciasis elimination with ivermectin treatment is feasible in some endemic foci in Africa, and this has already introduced a new paradigm for onchocerciasis control in the continent. Although this first evidence does not yet imply that elimination with ivermectin will be possible everywhere in Africa, the principle of elimination has been established. It now becomes a priority to evaluate in all onchocerciasis control programs in Africa their impact on onchocerciasis infection and transmission, and their progress towards elimination endpoints. The board of APOC has already acted upon the preliminary results of this study and adopted an additional objective for APOC to “develop the evidence base on when and where ivermectin treatment can be stopped, and provide guidance to countries on how to prepare for and evaluate cessation of treatment where feasible” [55]. APOC has already started to systematically collect epidemiological data on the impact of large-scale ivermectin treatment on onchocerciasis infection in different countries, focusing first on areas with the highest pre-control endemicity levels and different vector species.

When large scale ivermectin treatment started in 1987, it was not known if it would ever be possible to stop treatment. The present study has provided the first evidence that this is possible and that onchocerciasis elimination can be a realistic target, also in endemic areas in Africa.

## Supporting Information

Alternative Language Abstract S1 French translation of the abstract by Laurent Yaméogo.
(0.02 MB DOC)

Checklist S1 STROBE checklist.
(0.09 MB DOC)
