# Supplementary material for: Feasibility of Onchocerciasis Elimination with Ivermectin Treatment in Endemic Foci in Africa: First Evidence from Studies in Mali and Senegal
Source: PLoS Negl Trop Dis. 2009 Jul 21;3(7):e497. doi: 10.1371/journal.pntd.0000497 (PMC2710500; doi:10.1371/journal.pntd.0000497)
Supplement: Alternative Language Abstract S1 — French translation of the abstract by Laurent Yaméogo. (0.02 MB DOC) [file pntd.0000497.s001.doc]

**Faisabilité de l’élimination de l’onchocercose par le traitement à l’ivermectine dans des foyers endémiques en Afrique : première évidence établie à partir d’études menées au Mali et au Sénégal**

**Contexte**

Le traitement de masse à l'ivermectine est une stratégie bien établie pour lutter contre l'onchocercose en tant que problème de santé publique, mais l’on ne sait pas s’il peut également interrompre la transmission et éliminer le parasite dans les foyers endémiques en Afrique où les vecteurs sont très efficaces. C’est ainsi qu’une étude longitudinale a été conduite dans trois foyers hyperendémiques au Mali et au Sénégal où des traitements annuels ou semestriels à l'ivermectine ont été effectués pendant 15 à 17 ans, afin d'évaluer les niveaux résiduels de l'infection et de la transmission, et tester la faisabilité de l’arrêt, sans risques, du traitement à l'ivermectine dans les zones d'étude.

**Méthodologie/principaux résultats**

Des enquêtes parasitologiques par biopsie cutanée ont été effectuées dans 126 villages où 17.801 personnes ont été examinées. La prévalence en terme de microfiladermie était < 1% dans chacun des trois foyers. Au total, 150,500 simulies ont été collectées et analysées en vue de détecter la présence de larves d’*O. volvulus* en utilisant une sonde ADN spécifique, et les taux d'infectivité du vecteur étaient en dessous de 0,5 simulies infectieuses pour 1.000 simulies analysées. A l’exception d’une sous-section d'un des foyers, tous les indicateurs de transmission et d’infection étaient en dessous des seuils indiqués pour l'élimination. Le traitement a donc été suspendu dans des zones tests couvrant 5 à 8 villages dans chaque foyer. Les évaluations effectuées 16 à 22 mois après le dernier traitement dans les zones tests comprenaient l'examen de 2.283 personnes par la méthode de biopsie cutanée et le test de pansement à la DEC, et l’analyse de 123,000 simulies capturées. Aucune personne ni simulie infectée n'a été trouvée dans les zones tests, et les taux d'infectivité du vecteur sur d'autres points de captures étaient < 0,2 simulies infectieuses pour 1.000.

**Conclusion/importance**

L'étude a fourni la première évidence empirique que l'élimination de l'onchocercose par le traitement à l'ivermectine est faisable dans quelques foyers endémiques en Afrique. Bien que d'autres études soient nécessaires pour déterminer dans quelle mesure ces résultats peuvent être extrapolés à d'autres zones endémiques en Afrique, le principe de l'élimination a été établi. Le programme africain de lutte contre l'onchocercose a ainsi adopté un objectif additionnel pour évaluer les progrès vers l’atteinte de l'élimination dans tous les projets de lutte contre l'onchocercose et pour guider des pays pour l’arrêt des traitements là où cela est faisable.
